# Healthy China 2030: how to control the rising trend of unintentional suffocation death in children under five years old

**DOI:** 10.1186/s12887-020-02281-9

**Published:** 2020-08-13

**Authors:** Fanjuan Kong, Lili Xiong, Aihua Wang, Donghua Xie, Jian He, Jinping Su, Kui Wu, Zhiyu Liu, Hua Wang

**Affiliations:** 1 Department of Information Management, Hunan Provincial Maternal and Child Health Care Hospital, 53 Xiangchun Road, Changsha, 410078 Hunan China; 2 Department of Pediatrics, Hunan Provincial Maternal and Child Health Care Hospital, 53 Xiangchun Road, Changsha, 410078 Hunan China; 3 NHC Key Laboratory of Birth Defect for Research and Prevention, Hunan Provincial Maternal and Child Health Care Hospital, 53 Xiangchun Road, Changsha, 410078 Hunan China

**Keywords:** Unintentional suffocation, Children under five years old, Children under 1 -year-old, Children aged 1 to 4 years, Death

## Abstract

**Background:**

To investigate the occurrence frequency, changing trends, and epidemiological distribution of unintentional suffocation in children under 5 years old.

**Methods:**

The data were collected from the Maternal and Child Health Surveillance system from 2009 to 2018. The cause of death was classified by ICD-10. Data on unintentional suffocation death were calculated according to the characteristics of the population, time, space, cause of death and medical treatment, and constituent ratio were calculated.

**Results:**

The mortality rate of children under 5 years old showed a downward trend, but the mortality of unintentional suffocation initially decreased and then increased. The death rate of unintentional suffocation in children less than 1-year-old was much higher than that in children aged 1 to 4 years old. The death rate of unintentional suffocation was higher in boys than in girls, and the rate was higher for rural children than for urban children. The number of low-weight and pre-term infants in the group under 1-year-old was significantly higher than that in the group of 1–4 years old. Children under 1-year-old are more likely to die at home than children aged 1 to 4 years old, and a higher proportion of younger children did not receive treatment. More than 80% of children under the age of 5 go untreated because it was too late to go to the hospital.

**Conclusion:**

For areas and populations with a high incidence of unintentional suffocation, we suggest that priorities should include prevention, the development of a safe environment, strengthened prevention, the development of safety habits, and the popularization of first aid knowledge.

## Background

Child injury is a significant global public health problem, and more than 98% of child injury deaths occur in developing countries [1]. Injuries mainly include road traffic injuries, suicide, falls, and drowningdrowning [2]. Life expectancy in developing countries is reduced by 1.19 years due to injury [3]. In China [4], the proportion of injury deaths among 0–14 years of children in China rose from 18.69% in 2004 to 21.26% in 2011. Injury is also the leading cause of death among children aged 1 to 4 years old [2]. In China, 14.6% of deaths among children under 5 years of age are due to injuries [5].

Injuries, like diseases, can be recognized, prevented, and controlled [6–8]. The mortality rate of children under 5 years of age in China dropped from 61.0 per thousand in 1991 to 8.4 per thousand in 2018, a decrease of 86.2% [9]. Unintentional suffocation constitutes the most significant proportion of injury deaths among children under the age of 5 in China [9–11]. However, the current research focuses on either major-specific injuries, such as traffic accidents or drowning, or the entire age group of children. A previous study investigated under-five mortality from unintentional suffocation in the Chinese population from 2006 to 2016 and reported a total of 2937 cases from 161 surveillance points [12], which covered 161 surveillance points in China and included a sample size of 2937 cases. This study analyzed the incidence and causes of unintentional suffocation death and compared the death rates of different age groups, men and women, and urban and rural areas.

This present study covers all areas of Hunan Province from 2009 to 2018 for population-wide surveillance and includes a sample size of 4933. A critical supplement to previous research is to analyze the treatment and causes of death of children in different age groups. To reduce the unintentional suffocation mortality rate of children under 5 years old in the province and improve the health level of children in an attempt to truly implement the goal of “healthy China 2030” [13]. It is necessary to investigate and analyze the death situation and the changing trend of this part of the population.

## Method

### Data source

**Fig. 1:**
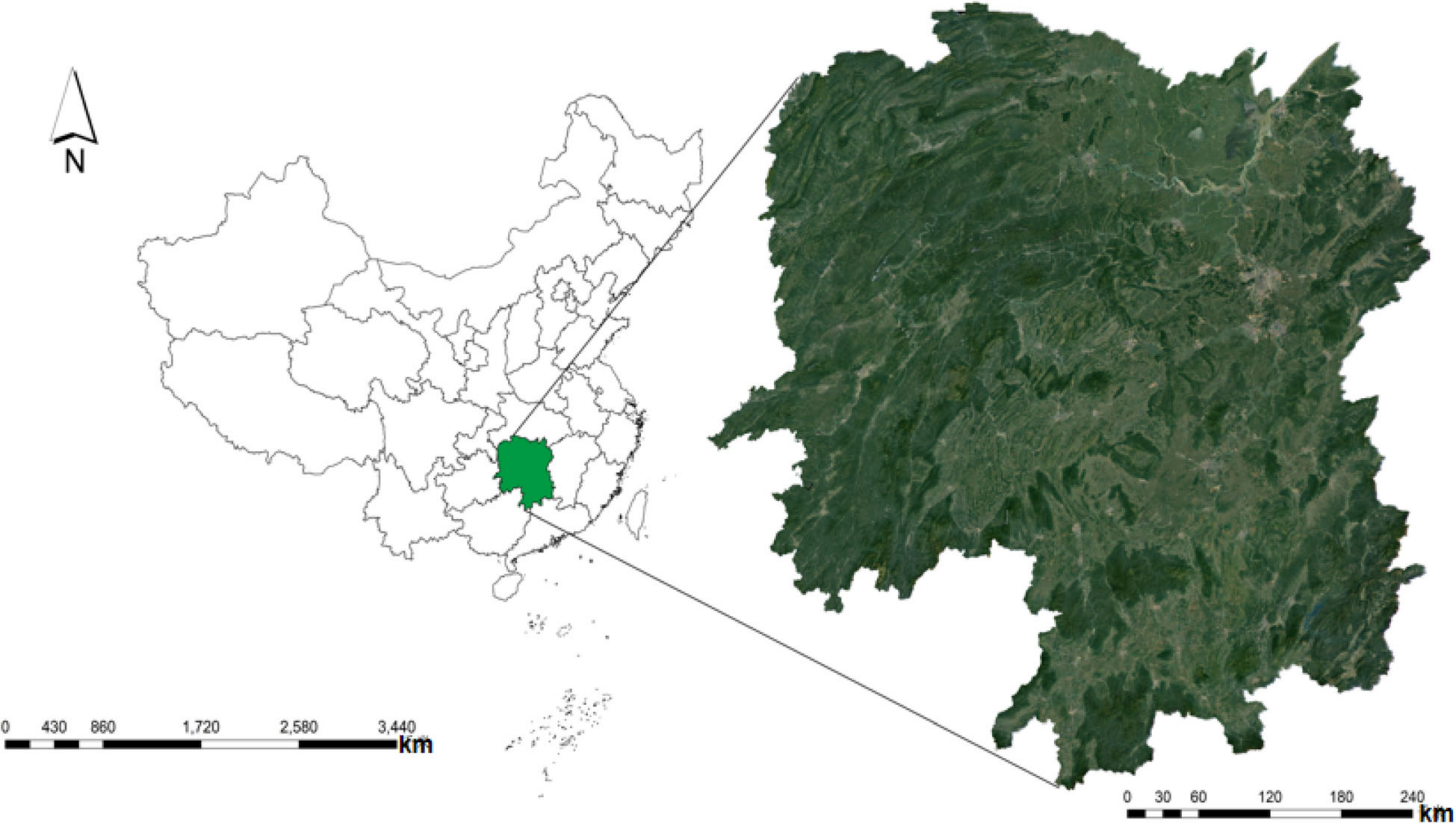
The geographical location of Hunan Province in China.(GIS10.2, The URL is https://user.qzone.qq.com/327532957/infocenter)

Hunan Province (Fig. 1.The URL is https://user.qzone.qq.com/327532957/infocenter) is located in the hinterland of southeast China and the middle reaches of the Yangtze River, which is the bridge between the eastern coastal provinces and the western inland provinces. The land area of the region is 211,800 km^2^, with mountains accounting for approximately half of the total area and plains, basins, hills, and water surfaces accounting for approximately half. The population of the province is approximately 73 million. The province has a mild climate and four distinct seasons, with sufficient heat and concentrated precipitation, changeable spring temperatures and drought in summer and autumn, a short severe cold period, and a long summer heat period.

The cause of death data comes from the maternal and child health surveillance system, which covers all the data on accidental injury deaths in Hunan Province and conducts the total population surveillance. The Chinese death Surveillance Program for Children under 5 requires that the root causes of deaths of children under 5 be coded according to the International Classification of Diseases (ICD-10). Between 2009 and 2018, a total of 59,880 children under 5 years of age died and 4933 cases of unintentional suffocation were identified. Unintentional suffocation refers to hypoxia and asphyxia caused by accidental causes, such as the baby being covered by a quilt, mother turning over and accidentally crushing to death, mother’s nipple blockage, and foreign body in the trachea.

China has established a child death reporting network and corresponding monitoring system centered on maternal and child health care institutions, with special personnel at all levels responsible for the collection, collation and preservation of data. All deaths are required to fill in the “Child death report Card” and use paper reports and direct network reports in parallel.

### Quality control

Child death information is reported, verified and collected step by step by the village and township health center, the district and county maternal and child health institutions, the municipal maternal and child health hospital, the provincial maternal and child health hospital, the China Disease Prevention and Control Center and the Maternal and Child Health Center. All levels check the logic, completeness, and correctness of the collected child death report cards. Random spot checks are conducted annually by the state at the provincial level, twice a year at the municipal level, and once a quarter at the district and county levels. Child death information from family planning, public security, civil affairs, disease control and control departments (“National Population and Death Information Registration and Management system”) and medical institutions is checked and supplemented.

### Data reporting method

The death information was reported through the Hunan Maternal and Child Health Surveillance Information system according to the requirements of the China Maternal and Child Health Surveillance Information system.

### Statistical methods

The fundamental cause of death was judged, coded and classified by ICD-10, and the data on the cause of death was analyzed by total mortality, disease-specific mortality, age-specific mortality and the composition of death causes and statistically analyzed using the SPSS17.0 software package. The primary statistical analysis methods were the chi-square test and chi-square trend test. We used GIS10.2 software to make a map of Hunan Province.

## Results

From 2009 to 2018, the maternal and child health surveillance system showed that there were 7,942,123 live births, 59,880 deaths of children under 5 years old, and 4933 children died of unintentional suffocation. The mortality rate of children under 5 years old showed a downward trend from 10.9‰ in 2009 to 5.3‰ in 2018, decreased by 51.4%.The death rate of unintentional suffocation in children under 5 years old decreased from 90.8/100000 in 2009 to 45.4/10000 in 2017, decreased by 50%.But the death rate of unintentional suffocation in 2018 was higher than that in previous years. There were significant differences in child mortality and unintentional suffocation mortality over the years (P<0.001). The proportion of unintentional suffocation deaths in the number of children under the age of 5 showed a fluctuating upward trend from 8.4% in 2009 to 11.7% in 2018, increased by28.2% Table 1.

**Table 1:** Death and unintentional suffocation of children under five years of age from 2009 to 2018

Year	Number of live births	Children under 5 years old		Unintentional suffocation		Percentage of deaths in children under 5 years of age (%)
		N	Mortality Rate (per 1000 persons)	N	Mortality Rate (per 100,000 persons)	
2009	781,026	8482	10.9	709	90.8	8.4
2010	797,902	8636	10.8	584	73.2	6.8
2011	805,360	7509	9.3	586	72.8	7.8
2012	838,974	7343	8.8	642	76.5	8.7
2013	821,345	5747	7.0	466	56.7	8.1
2014	780,572	5087	6.5	316	40.5	6.2
2015	781,066	4791	6.1	404	51.7	8.4
2016	795,399	4435	5.6	405	50.9	9.1
2017	834,955	4084	4.9	379	45.4	9.3
2018	705,524	3766	5.3	442	62.6	11.7
**Total**	**7,942,123**	59,880	7.5	4933	62.1	8.2
**χ**^**2**^		4729.545		291.504		
**P**		< 0.001		< 0.001		

**Fig. 2:**
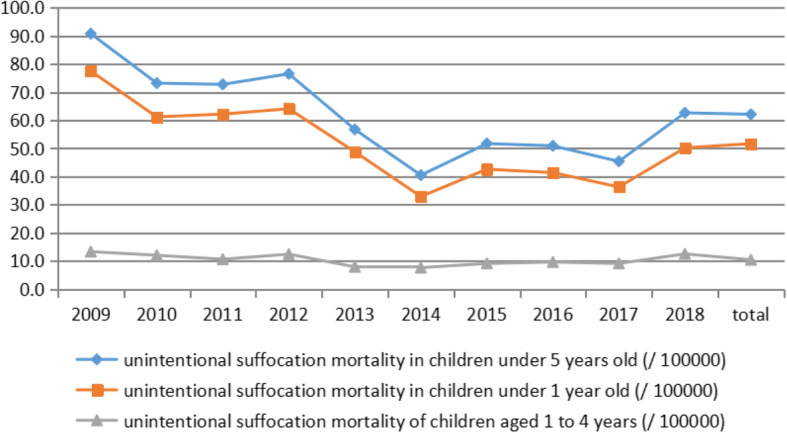
Comparison of unintentional suffocation mortality among children of different age groups

The number of unintentional suffocation deaths among children under 5 years old was 4933, of which 4109 (83.3%) were under 1-year-old and 824 (16.7%) were 1–4 years old. The unintentional suffocation mortality rate of children under 1-year-old decreased from 77.5/100000 in 2009 to 50.2/100000 in 2018, decreased by 35.2%.The mortality rate of unintentional suffocation in children aged 1 to 4 years fluctuated around 10/100000. The mortality rate of unintentional suffocation in children under 1 -year-old was significantly higher than that in children aged 1–4 years old, which was about 5 times higher than that in children aged 1–4 years old. As shown in Table 2 and Fig. 2.

**Table 2:** Unintentional suffocation deaths of children less than 1 year old and children aged 1 to 4 years old

Year	Number of infant deaths	Unintentional suffocation of infants under 1 year old			Number of deaths of children aged 1 to 4 years old	Unintentional suffocation in children aged 1 to 4 years old		
		N	Mortality rate (per 100,000 persons)	Percentage of infant deaths (%)		N	Mortality Rate (per 100,000 persons)	Percentage of deaths among children aged 1 to 4 years old
2009	6058	605	77.5	10.0	2424	104	13.3	4.29
2010	5868	488	61.2	8.3	2768	96	12	3.47
2011	5138	501	62.2	9.8	2371	85	10.6	3.58
2012	4803	538	64.1	11.2	2540	104	12.4	4.09
2013	3851	401	48.8	10.4	1896	65	7.9	3.43
2014	3277	257	32.9	7.8	1810	59	7.6	3.26
2015	3117	333	42.6	10.7	1674	71	9.1	4.24
2016	2825	329	41.4	11.6	1610	76	9.6	4.72
2017	2592	303	36.3	11.7	1492	76	9.1	5.09
2018	2367	354	50.2	15.0	1399	88	12.5	6.29
**Total**	**39,896**	**4109**	**51.7**	**10.3**	19,984	**824**	10.4	4.12

For infants under1-year-old, the mortality rate of unintentional suffocation was 55.7/100000 in rural areas and 44.2/100000 in urban areas. The death rate of unintentional suffocation was 55.1/100000 in boys and 47.6/100000 in girls. For children aged 1–4 years old, the mortality rate of unintentional suffocation was 10.5/100000 in rural areas and 9.6/100000 in urban areas. The death rate of unintentional suffocation was 12.8/100000 in boys and 7.6/100000 in girls. The infants under 1-year-old in urban and rural areas and males and females were higher than those in 1–4 years old group (*P* < 0.001). As shown in Table 3.

**Table 3:** Comparison of the incidence of Unintentional suffocation between urban and rural areas and between men and women

Indicators	Number of live births^a^	< 1 year old		1–4 years old		χ^2^	*P*
		N	Mortality rate (per 100,000 persons)	N	Mortality rate (per 100,000 persons)		
Rural	5,220,568	2906	55.7	550	10.5	1606.648	< 0.001
Urban	2,721,400	1203	44.2	262	9.6	604.587	< 0.001
Male	4,232,951	2331	55.1	541	12.8	1116.02	< 0.001
Female	3,709,017	1766	47.6	281	7.6	1077.593	< 0.001

^a^There were 155 cases of lack of live birth information

There were 2826 cases (68.7%) of infants under 1-year-old in the first and fourth quarters, 361 cases (8.8%) of low birth weight infants and 287 cases (7.0%) of premature infants. 1906 (46.4%) died at home, 614 (12.1%) died on the way to the hospital, 2942 (71.6%) did not receive treatment, 2406 (81.8%) had no time to go to the hospital, and 65.2% of the deaths were postmortem. Most of the children aged 1 to 4 are full-term and normal-weight babies. Two hundred forty-five children (29.7%) died at home, 204 children (24.8%) died on their way to the hospital, and 510 children (61.9%) did not receive treatment because 430 children (84.3%) did not have time to go to the hospital. 54.4% of deaths were inferred postmortem. As shown in Table 4.

**Table 4:** Comparison of the basic situation and treatment of unintentional suffocation between children less than 1 year old and children aged 1 to 4 years old

Indicators	< 1 year old		1–4 years old		χ^2^	*P*
	N	Proportion(%)	N	Proportion(%)		
**1. Gender**						
Male	2331	56.7	541	65.7	22.486	< 0.001
Female	1766	43.0	281	34.1		
Missing	12	0.3	2	0.2		
**2. Urban and rural areas**						
Rural	2906	70.7	550	66.7	62.854	< 0.001
Urban	1203	29.3	262	31.8		
Missing	0	0	12	1.5		
**3. Quarter of death**						
First quarter	1037	25.2	275	33.4	122.605	< 0.001
Second quarter	541	13.2	199	24.2		
Third quarter	742	18.1	131	15.8		
Fourth quarter	1789	43.5	219	26.6		
**4. Household registration**						
Local household registration	4019	97.8	820	99.5	10.818	0.004
Non-local household registration	80	2	4	0.5		
Missing	10	0.2	0	0.0		
**5. Birth weight**						
< 2500 g	361	8.8	3	0.4	88.129	< 0.001
2500 g–4000 g	3635	88.4	816	99.0		
> 4000 g	56	1.4	1	0.1		
Missing	57	1.4	4	0.5		
**6. Gestational age**						
< 37 weeks	287	7.0	2	0.2	90.321	< 0.001
37–40 weeks	3583	87.2	811	98.4		
> 40 weeks	176	4.3	7	0.8		
Missing	63	1.5	4	0.6		
**7. Place of death**						
Hospital	867	21.1	224	27.2	181.765	< 0.001
On the way to see a doctor	499	12.1	204	24.8		
On the way home after transfer or treatment	614	14.9	146	17.7		
Home	1906	46.4	245	29.7		
Missing	223	5.5	5	0.6		
**8. Antemortem treatment**						
Hospitalization	502	12.2	94	11.4	252.243	< 0.001
Outpatient	326	7.9	210	25.5		
Untreated	2942	71.6	510	61.9		
Missing	339	8.3	10	1.2		
**9. Main reasons for not being treated or not seeking medical treatment**						
Economic difficulties	10	0.3	0	0	67.487	< 0.001
Traffic inconvenience	22	0.8	4	0.8		
Too late to take child to the hospital.	2406	81.8	430	84.3		
Parents thought that the condition was not serious.	31	1.1	2	0.4		
Other	114	3.9	12	2.4		
Missing	359	12.1	62	12.1		
**10. Diagnostic basis of cause of death**						
Pathological autopsy	11	0.3	5	0.6	38.556	< 0.001
Clinical	1371	33.4	364	44.2		
Postmortem inference	2679	65.2	448	54.4		
Missing	48	1.1	7	0.8		

## Discussion

The main results are as follows. (1) The mortality rate of children under 5 years old showed a downward trend, but the death rate of unintentional suffocation showed an upward trend, and the proportion of unintentional suffocation death in children under 5 years old also increased. (2) The unintentional suffocation mortality rate of children under 1-year-old (51.7/100000) was much higher than that of 4-year-old children (10.4/100000). Children under 1-year-old were more likely to die at home from unintentional suffocation than children aged 5 years old, and more of the younger children did not receive treatment. (3) The unintentional suffocation mortality rate of boys and children in rural areas was higher than that of girls and children in urban areas.

The death rate of unintentional suffocation has changed from a decline to an upward trend, which is consistent with the direction of the United States [14]. The proportion of unintentional suffocation deaths to the number of children under 5 years old is also on the rise, which is consistent with a national study [10]. Most unintentional suffocation deaths (83.3%) were children under 1-year-old, which is consistent with reports from China [12], the United States [15], Canada [16] and Japan [17]. Children younger than 1-year-old cannot yet walk, and their injuries are mainly due to the negligence or abuse of their parents, while children aged 1–4 years old demonstrate hyperactivity, curiosity and exploration, resulting in many injuries to themselves [18].

The unintentional suffocation mortality rate of boys and rural children is higher than that of girls and urban children, which is consistent with previous studies [12, 19, 20]. Especially among children over 1-year-old, boys tend to be more impulsive and active and engage in riskier behaviors than girls [21]. The frequency of child injuries in underdeveloped communities in Pakistan is three times higher than that in developed cities [22]. The difference in injuries between rural and urban areas of China increases significantly with time [23], which may be related to the living environment, health awareness, medical facilities and inconvenient transportation in underdeveloped rural areas. In 2019, a total of 17.787 million migrant workers in Hunan left (https://www.anhui365.net/PostCenter/ThreadDetail/id/8761234) and approximately 700,000 children stayed at home with their grandparents [24], which may lead to inadequate care of these left-behind children. The study [25] have shown that the childhood injury rate of left-behind children in rural areas (33.5%) is significantly higher than that of non-left-behind children (28.6%).

The situation for children who die of unintentional suffocation is not optimistic. In the first and fourth quarters, the number of unintentional suffocation deaths was higher than that in the second and third quarters, which may be related to lower temperatures in the first and fourth quarters, thicker quilts for sleeping at night and being pinned down when sleeping with parents. Low weight and non-term infants were concentrated in children under 1-year-old, which may be related to immature organs, imperfect sucking and swallowing function, small stomach capacity, lack of digestive enzymes, poor absorption and digestion ability, and so on. Nearly 50% of children under the age of one died at home, 24.8% of children aged 1 to 4 died on their way to the hospital, and more than 80% of the children did not see a doctor because they did not have time to go to the hospital. This situation may be related to the rapid occurrence of unintentional suffocation and the short time of death. There are many rural and mountainous areas in Hunan Province, and the accessibility of medical services needs to be improved. The prevention of unintentional suffocation is the fundamental measure. Many parents do not know how to provide first aid on the spot, and they often panic when their children have accidents and do not give first aid treatment [26]. In China, the lack of standardized procedures for emergency rescue often results in the inappropriate or incorrect medical treatment of trauma and failure to stabilize patients before hospital admission [27].

The United Nations has incorporated injury prevention into the Sustainable Development goals action plan [28]. In 2008, a study in the Lancet reported on “injury-related deaths in China, a public health problem that has not been fully recognized” [29]. In 2011, China issued the Program for the Development of Chinese Children (2011–2020) [30], which called for a nearly 17% reduction in injury-related mortality among children under the age of 18. The outline of the Healthy China 2030 Plan [13] aims to establish a comprehensive injury monitoring system and formulate guidelines and standards for strengthening injury prevention and intervention, reducing traffic injuries and drowning, and preventing poisoning. These policies can play a specific role in reducing the occurrence of injuries. Nevertheless, the data from our province in the past 10 years show that the death rate of unintentional suffocation has an upward trend. Compared with developed countries such as the United States [31] and the United Kingdom [32], China lacks precise and specific action plans for child injury prevention.

The primary factor chain of an accidental injury is “no foresight consciousness—no preventive measures—no skill learning—no effective first aid”. According to the incidence, individual characteristics, urban and rural distribution, cause analysis and treatment of accidental injury death, the following prevention and control measures are proposed.

First, safe feeding care should be provided. The critical issue is to improve the feeding and nursing of infants and young children to prevent the occurrence of unintentional suffocation. Guardians need to master correct nutritional knowledge (e.g., feeding posture, feeding volume), especially premature infants, low birth weight infants and other at-risk children. It is necessary to pay attention to sleep care (sleep posture, bedding thickness, and weight). Children aged 1 to 4 years old have a high risk of inhalation suffocation [12]. Attention should be paid to the inhalation of foreign bodies in the respiratory tract and the management of nuts, beans and buttons as dangerous substances to avoid exposure to young children.

The second proposal is to build a safe environment and strengthen the management of the children’s living environment. Ribas Rde et al. [33] shows that the majority (61.7%) of accidental injuries occur in or near the home, and appropriate preventive measures can reduce the risk by 26%. Most unexplained infant deaths are potentially preventable and occur in highly dangerous sleep environments [34]. The safest way for infants to sleep is on their backs, on an unshared sleep surface, in a crib or bassinet in the caregivers’ room, and without soft bedding (e.g., blankets, pillows, and other soft objects) in their sleep area [35].

The third suggestion is to popularize the knowledge of first aid. Because of the sudden and unpredictable nature of accidental death, the on-the-spot rescue of accidental death is very important. A useful primary aid measure is the last line of defense to reduce death or disability. The experience of first aid is widely publicized in a variety of ways, such as providing common first aid knowledge, such as emergency handling of foreign body inhalation and cardiopulmonary resuscitation, into brochures, children’s songs, collective rap songs, dance, and demonstration videos. to improve caregivers’ awareness of injuries and their ability to deal with emergencies. In particular, it is necessary to involve maternal and child health care institutions with Chinese characteristics and make use of the three-level network of child health care for publicity and education.

A few factors limited to this study. First, our questionnaire was a retrospective survey with retrospective bias. However, our inquiry was conducted as early as possible to minimize information bias, and data quality control was conducted at all levels of provinces, cities, and counties every year. Second, our questionnaire did not collect information about children’s unintentional suffocation exposure, such as appropriate time, activity, and risk factors. We did not conduct a detailed analysis to credibly explain recent changes in mortality. Third, the contents of the unintentional suffocation death case card may be filled incorrectly or omitted. By setting logical detection and required options, and all levels of on-site quality control, our data quality has been guaranteed to a certain extent.

## Conclusion

In conclusion, we report a decline in mortality among children under 5 years of age from 2009 to 2018, but unintentional suffocation deaths initially decreased and then increased. The unintentional suffocation mortality rate of infants under 1-year-old was 51.7/100000, which was much higher than that of children aged 1 to 4 years old (10.4/100000). The mortality rate of unintentional suffocation in boys and rural areas was higher than that in girls and urban regions. Infants under 1-year-old were more likely to die at home than children aged 1 to 4 years old, and a higher proportion of younger children did not receive treatment. Targeted prevention strategies should be adopted to advocate prevention, build a safe environment, strengthen safety prevention, develop safety habits, popularize first aid knowledge, and curb the rising trend of unintentional suffocation mortality.

## Data Availability

No data are available. The cost estimates for this study were obtained under license and are not available for sharing.
